# Gender balance in WHO panels for guidelines published from 2008 to 2018

**DOI:** 10.2471/BLT.18.226894

**Published:** 2019-05-28

**Authors:** Meghan A Bohren, Dena Javadi, Joshua P Vogel

**Affiliations:** a Gender and Women’s Health Unit, Centre for Health Equity, Melbourne School of Population and Global Health, The University of Melbourne, Carlton, Victoria, 3053 Australia.; b Alliance for Health Policy and Systems Research, World Health Organization, Geneva, Switzerland.; c Burnet Institute, Melbourne, Australia.

## Abstract

**Objective:**

To assess the gender composition of guideline contributors for all World Health Organization (WHO) guidelines published from 2008 to 2018.

**Methods:**

We searched for guidelines in the WHO Guideline Review Committee database. We extracted data about the guidelines (title, publication year) and individuals participating (name, role, gender). Guideline roles included: member or chair of guideline development group, WHO steering group, external reviewer or methodologist. We used descriptive statistics to analyse gender composition for each role and the proportion of guideline development group members and chairs who were female.

**Findings:**

We included 230 guidelines involving 13 329 individuals: 219 guidelines (95.2%) reported a guideline development group (4912 individuals). More group members were male (2606; 53.1%) than female (2241; 45.5%). The median proportion of female members per guideline was 47.1% (interquartile range: 35.7–56.3). Half of the guidelines (110; 50.2%) had a development group composed of 40.1–60% females and 75 guidelines (34.2%) had ≤ 40% females in the group. From 2016 to 2018, there were some improvements: one quarter of groups were composed of ≤ 40.0% females in 2016 and 2017, and this reduced to 9.1% in 2018. Among 243 group chairs, 145 (59.7%) were male and 96 (39.5%) were female.

**Conclusion:**

Participation on a guideline panel is a prestigious leadership role in global health. The under-representation of women across most WHO guideline roles shows that inequalities persist even where standards and policies call for gender balance. Attention can be shifted to strengthening accountability mechanisms and understanding the root causes of this imbalance.

## Résumé

**Objectif:**

Évaluer la composition hommes-femmes des contributeurs aux directives de l'Organisation mondiale de la Santé (OMS) publiées de 2008 à 2018.

**Méthodes:**

Nous avons recherché des directives dans la base de données du Comité d'évaluation des directives de l'OMS. Nous en avons extrait des données concernant les directives (titre, année de parution) et les personnes qui y ont participé (nom, rôle, sexe). Les rôles étaient les suivants: membre ou président de groupe d'élaboration de directives, groupe de pilotage de l'OMS, réviseur externe ou méthodologiste. Nous avons utilisé des statistiques descriptives pour analyser la composition hommes-femmes de chaque rôle et la part de femmes membres ou présidentes de groupes d'élaboration de directives.

**Résultats:**

Nous avons étudié 230 directives sur lesquelles étaient intervenues 13 329 personnes: 219 directives (95,2%) mentionnaient un groupe d'élaboration de directives (4912 personnes). Ces groupes comptaient plus d'hommes (2606; 53,1%) que de femmes (2241; 45,5%) parmi leurs membres. La proportion médiane de femmes membres par directive était de 47,1% (intervalle interquartile: 35,7–56,3). La moitié des directives (110; 50,2%) avaient été élaborées par un groupe composé de 40,1% à 60% de femmes et 75 directives (34,2%) comptaient ≤ 40% de femmes dans le groupe. Certaines améliorations ont été observées entre 2016 et 2018: un quart des groupes était composé de ≤ 40,0% de femmes en 2016 et 2017, proportion qui était passée à 9,1% en 2018. Sur 243 présidents de groupes, 145 (59,7%) étaient des hommes et 96 (39,5%) des femmes.

**Conclusion:**

La participation à un panel sur les directives dans le domaine de la santé mondiale est un rôle prestigieux. La sous-représentation des femmes dans la plupart des rôles liés aux directives de l'OMS montre que des inégalités persistent, alors même que des normes et des politiques appellent à un équilibre hommes-femmes. Il conviendrait de renforcer les mécanismes de responsabilisation et de comprendre les causes profondes de ce déséquilibre.

## Resumen

**Objetivo:**

Evaluar la composición por género de los colaboradores de las directrices de la Organización Mundial de la Salud (OMS) publicadas entre 2008 y 2018.

**Métodos:**

Se realizaron búsquedas de directrices en la base de datos del Comité de examen de las directrices de la OMS. Se extrajeron los datos sobre las directrices (título, año de publicación) y los individuos participantes (nombre, función, sexo). Las funciones de las directrices incluían: miembro o presidente del grupo de desarrollo de directrices, grupo directivo de la OMS, revisor externo o metodólogo. Utilizamos estadísticas descriptivas para analizar la composición de género para cada función y la proporción de miembros y presidentes de grupos de desarrollo de directrices que eran mujeres.

**Resultados:**

Se incluyeron 230 directrices con 13 329 personas: 219 directrices (95,2 %) informaron un grupo de desarrollo de directrices (4 912 personas). Más miembros del grupo eran hombres (2 606; 53,1 %) que mujeres (2 241; 45,5 %). La proporción media de miembros femeninos por directriz fue del 47,1 % (rango intercuartil: 35,7-56,3). La mitad de las directrices (110; 50,2 %) tenían un grupo de desarrollo compuesto de 40,1-60 % de mujeres y 75 directrices (34,2 %) tenían ≤ 40 % de mujeres en el grupo. De 2016 a 2018, hubo algunas mejoras: una cuarta parte de los grupos estaban compuestos por un ≤ 40,0 % de mujeres en 2016 y 2017, y esto se redujo al 9,1 % en 2018. De los 243 presidentes de grupos, 145 (59,7 %) eran hombres y 96 (39,5 %) mujeres.

**Conclusión:**

La participación en un panel de directrices es una función de liderazgo prestigiosa en la salud mundial. La escasa representación de las mujeres en la mayoría de las funciones de las directrices de la OMS muestra que las desigualdades persisten incluso cuando las normas y las políticas exigen el equilibrio entre los géneros. Se puede prestar atención al fortalecimiento de los mecanismos de rendición de cuentas y a la comprensión de las causas profundas de este desequilibrio.

## ملخص

الغرض تقييم التكوين من الجنسين للمساهمين بالمبادئ التوجيهية في كل المبادئ التوجيهية لمنظمة الصحة العالمية (WHO) المنشورة خلال الفترة من 2008 إلى 2018.

الطريقة قمنا بالبحث عن المبادئ التوجيهية في قاعدة بيانات لجنة مراجعة المبادئ التوجيهية لمنظمة الصحة العالمية. وقمنا باستخراج بيانات عن المبادئ التوجيهية (العنوان، وسنة النشر)، والأفراد المشاركين (الاسم، والدور، والجنس). شملت أدوار المبادئ التوجيهية: عضو أو رئيس مجموعة تنمية المبادئ التوجيهية، أو مجموعة توجيهية بمنظمة الصحة العالمية، أو مراجع خارجي، أو عالم مناهج. قمنا باستخدام الإحصاءات الوصفية لتحليل التكوين من الجنسين لكل دور، ونسبة أعضاء مجموعة تطوير المبادئ التوجيهية، والرؤساء الذين كانوا من الإناث.

النتائج قمنا بتضمين 230 من المبادئ التوجيهية تشمل 13329 فردًا: أبلغ 219 من المبادئ التوجيهية (95.2%) عن مجموعة لتطوير المبادئ التوجيهية (4912 فردا). المزيد من أعضاء المجموعات كانوا ذكوراً (2606؛ 53.1%) أكثر من الإناث (2241؛ 45.5%). كانت النسبة المتوسطة للأعضاء من الإناث لكل مبدأ توجيهي 47.1% (المدى الربيعي: 35.7–56.3). نصف المبادئ التوجيهية (110؛ 50.2%) كان لها مجموعة تطوير تتكون من الإناث بنسبة من 40.1 إلى 60%، أما 75 من المبادئ التوجيهية (34.2%) كانت تتضمن 40% أو أقل من الإناث في المجموعة. خلال الفترة من 2016 إلى 2018، كانت هناك بعض التحسينات: تكونت ربع المجموعات من 40% أو أقل من الإناث في عامي 2016 و2017، وانخفضت هذه النسبة إلى 9.1% في عام 2018. ومن بين 243 من رؤساء المجموعات، كان هناك 145 (59.7%) من الذكور، و96 (39.5%) من الإناث.

الاستنتاج تعتبر المشاركة في فرق المبادئ التوجيهية دوراً قيادياً بارزاً في قطاع الصحة العالمي. إن التمثيل المنخفض للمرأة عبر معظم أدوار المبادئ التوجيهية بمنظمة الصحة العالمية، يشير إلى استمرار حالات عدم المساواة حتى عندما تدعو المعايير القياسية والسياسات إلى التوازن بين الجنسين. يمكن تحويل الانتباه إلى دعم آليات المساءلة، واستيعاب الأسباب الجذرية لهذا الخلل في التوازن. ****

## 摘要

**目的:**

旨在评估 2008-2018 年间发布的所有世卫组织指南编撰者的性别构成。

**方法:**

我们搜索了世卫组织指南评审委员会数据库中的指南。提取了有关指南（标题、出版年份）和各参与者（姓名、职务、性别）的数据。指南小组职务包括：指南编撰小组成员或主席、世卫组织指导小组、外部审查员或方法学家。我们采用描述性统计数据分析每个职务的性别构成，以及指南编撰小组成员和主席中的女性比例。

**结果:**

我们纳入了 230 份指南，涵盖 13329 人。其中 219 份指南 (95.2%) 由指南编撰小组 (4912 人) 讨论编撰。小组成员中的男性（2606；53.1%）多于女性（2241；45.5%）。每个指南编撰小组的女性成员比例中位数为 47.1%（四分位差：35.7-56.3）。一半指南（110；50.2％）的编撰小组由 40.1-60％ 的女性组成，75 份指南 (34.2％) 的编撰小组由≤40％ 的女性组成。从 2016 年至 2018 年，情况有所改善：2016 年和 2017 年，四分之一的小组由≤40.0% 的女性组成，而 2018 年这一比例下降至 9.1%。在 243 名小组主席中，有 145 名男性(59.7%)， 96 名女性(39.5%)。

**结论:**

参与指南专家组可在全球卫生领域内发挥举足轻重的领导作用。大多数世卫组织职能中女性代表不足表明，即使在标准和政策呼吁性别平衡的地方，不平等依然存在。需要将关注点转移到加强问责机制和了解不平等的根源上。****

## Резюме

**Цель:**

Оценка гендерного состава групп авторов рекомендаций Всемирной организации здравоохранения (ВОЗ), опубликованных на протяжении 2008–2018 гг.

**Методы:**

Авторы провели поиск рекомендаций в базе данных Комитета ВОЗ по пересмотру рекомендаций. Были извлечены данные о рекомендациях (название, год публикации) и лицах, участвовавших в их составлении (имя, должность, пол). Состав авторов включал следующие должности: члена или председателя группы разработчиков, члена руководящей группы ВОЗ, внешнего рецензента или методиста. Авторы использовали описательные статистические данные для анализа гендерного состава каждой должности и для выявления доли женщин среди членов или председателей групп разработчиков рекомендаций.

**Результаты:**

В рассмотрение было включено 230 рекомендаций, над которыми работали в общей сложности 13 329 человек. Для 219 рекомендаций (95,2%) был известен состав группы разработчиков (4912 человек). Мужчины преобладали в составе разработчиков (2606 (53,1%) против 2241 (45,5%) женщины). Медианная пропорция женщин из расчета на одно руководство составила 47,1% (межквартильный диапазон: 35,7–56,3). Половина рекомендаций (110; 50,2%) имели группу разработчиков, состоявшую из женщин на 40,1–60%, а 75 рекомендаций (34,2%) имели ≤ 40% женщин в группе разработчиков. В период с 2016 по 2018 год отмечалось некоторое улучшение ситуации: четверть групп имели в составе ≤ 40% женщин в 2016 и 2017 годах, а к 2018 году этот показатель сократился до 9,1%. Среди 243 председателей групп разработчиков 145 человек (59,7%) были мужчинами, а 96 человек (39,5%) — женщинами.

**Вывод:**

Участие в группе, разрабатывающей рекомендации, подтверждает престижное лидерское положение врача в мировом здравоохранении. Недостаточная представленность женщин на большинстве должностей среди разработчиков рекомендаций ВОЗ говорит о том, что неравенство по-прежнему существует даже там, где стандарты и правила призывают к гендерному равенству. Следует привлечь внимание к укреплению механизмов ответственного поведения и пониманию основных причин такого неравенства.****

## Introduction

Despite women making up most the health workforce globally,^1^ persistent gender inequalities have been documented in terms of pay, leadership opportunities, and management.^2^^,^^3^ For example, women often earn less for the same work compared to men,^2^^,^^3^ and are overlooked for leadership positions in global health.^3^^,^^4^ These harmful gender norms can lead to women leaving scientific fields more frequently than men,^5^^,^^6^ in addition to limiting diversity in innovation, thought and opportunity. Gender inequalities in the health workforce and global health leadership are now well documented.^7^^–^^12^ However, transformative approaches, that is, approaches that tackle root causes of gender inequality and reshape power relations, are needed to address gender inequalities in global health leadership today.^13^

The World Health Organization (WHO) establishes global standards for clinical and public health interventions and policies, and is perceived to be the key global authority for health. Recently, WHO has been criticized for the under-representation of women in some spheres of its work. For example, at the 70th World Health Assembly in 2017, only 31% of the 191 heads of Member State delegations were women.^14^ Likewise, at the start of the new Director-General’s mandate in July 2017, only 30% of Directors were women,^14^ and only 42% of WHO staff in professional categories were women.^15^ In October 2017, the Director-General appointed a senior leadership team, made up of 67% women (10/15). While this is a promising shift, gender imbalances persist throughout WHO’s staffing, which may impact its technical work globally. WHO acknowledged this in its 13th General Programme of Work: “WHO cannot work effectively on gender equality and health equity without turning the mirror upon itself.”^14^

One of WHO’s core functions is the development and implementation of evidence-based guidelines on a range of health topics. These guidelines are critical to support countries, health ministries, health systems and health-care providers to achieve the best health outcomes possible, while promoting the right to health for all people. The WHO Guideline Review Committee was established in 2007 to ensure that WHO guidelines are high-quality, evidence-based, and developed using a transparent process. The committee plays an oversight and quality assurance role in WHO guideline development, and their standards and procedures are described in the committee’s handbook.^16^ WHO guidelines are typically coordinated by a steering group of WHO staff, and supported by a guideline methodologist and systematic review team. Box 1 outlines the key contributors and their roles within WHO guideline development. The steering group is responsible for identifying independent external experts to voluntarily participate in a guideline development group, which reviews evidence and formulates recommendations and an external review group that peer-reviews the guideline before publication. The WHO steering group is also responsible for ensuring balance and representation within this group of experts.

Box 1 **Roles of contributors to WHO guideline development**

**WHO steering group:** defined as all WHO staff named in the guideline (whether from headquarters, regional or country offices). The role of the WHO steering group is to provide administrative support for guideline development, create the scope of the guideline and key questions (using the population, intervention, comparison, outcome format), and identify individuals to participate in the guideline development group, the methodologists and the external review group. The WHO steering group is also responsible for drafting recommendations based on guideline development group decisions, drafting the final guideline, and publishing and disseminating the guideline.

**Guideline development group:** includes members and chair(s) named in the guideline. Group members are experts external to WHO whose primary task is the development of evidence-based recommendations. Members also contribute to deciding the scope of the guideline and identifying key questions. Members are identified by the WHO steering group and are “selected to encompass the technical skills, diverse perspectives and geographic representation needed” for guideline development.^16^ The group is intended to be multidisciplinary and “balanced in terms of gender and geography.”^16^ Groups typically comprise 10 to 20 experts and usually meet at least once face-to-face, as well as via online seminars or teleconferences. The group chair is typically nominated by the WHO steering group and confirmed by the group members. The chair should also be an expert in consensus decision-making and evidence interpretation, and have no financial conflicts of interest.

**External review group:** can engage in different stages of the guideline process, including reviewing the scope and key questions at the beginning of the guideline process, and providing peer review of the draft guideline document. The group is typically composed of individuals interested or experienced in the guideline topic that would be affected by the recommendations (i.e. stakeholders) or methodologists.

**Guideline methodologist:** is an expert in the guideline development process and methods, including systematic reviews, grading of recommendations, assessment, development and evaluations, and knowledge translation. Guideline methodologists are involved throughout the guideline process and play a key role in ensuring that the guideline development group formulates evidence-informed recommendations in a transparent manner.

**Other:** depending on the guideline, other individuals may participate in the guideline meeting or guideline development process. This would include observers, who are other stakeholders present at the guideline development group meeting, such as individuals who work at nongovernmental organizations, advocacy groups, funders, target audiences and service-users. An observer’s role is strictly to observe; they do not participate in the formulation of recommendations or the consensus process. However, they may provide their opinions or input as necessary. We also classified other individuals or groups (that were not otherwise specified as any of the four groups above) as other, including (but not limited to) representatives of other United Nations agencies, international partners, technical advisory or resource groups and external resource experts.

WHO: World Health Organization. Source: World Health Organization Guideline Review Committee handbook.^16^

Participation on a WHO guideline panel is a prestigious leadership role in global health, bringing with it the responsibility of formulating evidence-based recommendations that can affect the health and well-being of global populations. Guideline development group members who are health professionals are recognized technical experts in their fields, while other members represent the views and values of groups that are directly affected by the recommendations, such as consumer representatives. Ensuring gender equitable representation within guideline panels is important for ensuring the inclusion of diverse perspectives and relevance of the guideline to the affected population. This requirement is reflected in the *WHO handbook for guideline development*, which explicitly states that the guideline development and external review groups should be multidisciplinary and “balanced in terms of gender and geography.”^16^ There are several potential impacts of gender imbalances on guideline panels, including potentially different assessments of strength of recommendations, attention given to non-clinical measures such as values and preferences, as well as management of the decision-making processes. Following informal observations of several WHO guideline panels, we hypothesized that women were under-represented. The aim of this analysis was therefore to assess the gender composition of WHO guideline panels for all published WHO guidelines over an 11-year period.

## Methods

We defined a WHO guideline as any document developed by WHO containing recommendations for clinical practice or public health policy, that was reviewed and approved by the WHO Guideline Review Committee and published by WHO.^16^ We used the handbook^16^ to predefine five roles for involvement in WHO guideline development (Box 1). The committee suggests that 10–20 guideline development group members per guideline is usually sufficient, but may vary depending on the scope of the guideline.^16^

### Data sources and eligibility

The committee maintains a database of approved and published WHO guidelines, and we requested all guideline documents approved by the committee from 2008 to 2018 inclusive from the secretariat of the committee. We cross-referenced this database with a book search of the PubMed® online database for “WHO guidelines approved by the guidelines review committee,” which lists all WHO guidelines published by year and identified by an international standard book number. These guideline documents typically include the name and institutional affiliation for each member of a guideline development group and other individuals directly involved in guideline development. Two authors independently reviewed each document to assess whether it met the definition of a WHO guideline and if individuals contributing to the guideline development were listed. In the event of disagreement on eligibility, we sought the opinion of a third author or clarification from the secretariat.

### Data extraction and management

We extracted data about the guideline (title, year of publication) and the individuals involved (name, role category and gender). We assigned one role category to each individual (guideline development group member, guideline development group chair, WHO steering group member, external review group member, methodologist or other). Gender was categorized as female, male, other (e.g. non-binary gender identification) or unknown.

We developed and pilot-tested a data extraction form on a sample of five guidelines. After reviewing the responses and updating the form, we then extracted the relevant data from all included guidelines (one form per guideline). A second author reviewed the data and disagreements were resolved through discussion or review by a third author (e.g. if one reviewer labelled gender unknown and another reviewer was able to identify the gender). Where necessary, we sought clarification on roles from WHO colleagues working in the technical unit responsible for guideline development. We identified gender based on information reported in guideline documents, either directly as male or female or indirectly via the title of the individual (e.g. Ms, Mr or Mrs) or gendered pronouns and titles mentioned in the document. Where gender was not specified, we conducted an internet search for the named individual via their institutional website or public online professional websites and platforms (e.g. LinkedIn, ResearchGate, Google Scholar). We merged all extracted data and performed a series of internal quality checks. We anonymized the database (replacing individual and guideline names with unique identifiers) for the final analysis. We categorized any missing data as unknown.

We did not seek ethical approval as this was an analysis of publicly available information, and we did not contact any individuals who contributed to the guideline. No information that could identify individuals or guidelines is reported in this paper or supporting data.

### Data analysis

We calculated descriptive characteristics for all included guidelines and individuals, and stratified individual-level data by role and gender. The primary outcome for assessing gender balance in each guideline was the proportion of the guideline development group (including members and chairs) who were female. This was calculated for all guidelines and reported by year. We conducted analyses using SPSS version 20 (IBM Corp., Armonk, United States of America) and Stata version 15 (StataCorp, College Station, USA). We followed the guidelines of the Preferred Reporting Items for Systematic Reviews and Meta-analyses checklist to improve the transparency of this analysis.

## Results

A total of 289 documents were identified by the WHO Guideline Review Committee secretariat and our database search. We excluded 59 documents that did not meet the definition of a WHO guideline (e.g. tools, manuals or summaries derived from WHO guidelines or guideline implementation products) or did not include any information about individuals involved in the development. We included 230 guidelines published from 2008 to 2018. Several of these documents (29 documents) were updates of previously published guidelines or consolidated guidelines.

The 230 guidelines included 13 329 individuals across all roles (Table 1); some individuals participated in more than one guideline. In total, 5998 individuals (45.0%) were female, 6940 (52.1%) were male and none were other genders (e.g. non-binary). For 391 (2.9%) individuals, gender was unable to be ascertained. This was typically due to a lack of personal information in the guideline documents (such as full name of the individual or institutional affiliation).

**Table 1:** Individuals contributing to WHO guideline development, by gender and role in 219 guidelines published, 2008–2018

Role	No. (%) of individuals^a^			
	Total	Female	Male	Unknown gender
**Guideline development group member (including chair)**	4 912 (36.9)	2 241 (45.6)	2 606 (53.1)	65 (1.3)
**Guideline development group chair**	243 (1.8)	145 (59.7)	96 (39.5)	2 (0.8)
**Guideline methodologist**	282 (2.1)	179 (63.5)	102 (36.2)	1 (0.4)
**WHO steering group member**	3 721 (27.9)	1 637 (43.9)	2 044 (54.9)	40 (1.1)
WHO headquarters staff	3 035 (22.8)	1366 (45.0)	1640 (54.0)	29 (1.0)
WHO regional or country office staff	686 (5.1)	271 (39.5)	404 (58.9)	11 (1.6)
**External review group staff**	2 355 (17.7)	1 045 (44.4)	1 279 (54.3)	31 (1.3)
**Other^b^**	2 059 (15.4)	896 (43.5)	909 (44.1)	254 (12.3)
**Total**	**13 329 (100.0)**	**5 998 (45.0)**	**6 940 (52.1)**	**391 (2.9)**

WHO: World Health Organization.

^a^ Column percentages do not total 100.0% because of sub-categories (guideline development group chairs are also guideline development group members, WHO headquarters and WHO regional or country offices are part of WHO staff) overlapping with categories.

^b^ Please see Box 1 for roles included in this group.

Note: Of 230 guidelines identified, we excluded 11 guidelines from this analysis because they did not explicitly name panel members in the guideline development group.

### Members and chairs

A total of 219 guidelines over 2008–2018 reported a guideline development group. We excluded 11 guidelines from the analysis of group composition because the group members were not clearly specified from other contributors (10 of these guidelines were published in 2009). The size of the guideline development group varied across guidelines. The median number of members per guideline was 19.0 (interquartile range, IQR: 15.0–25.5). Across all guidelines, 4912 individuals were identified as group members, including the chairs (Table 1). Of the total members, we ascertained 2241 (45.6%) as female and 2606 (53.1%) as male; for 65 (1.3%) we could not determine the gender. 

**Fig. 1:**
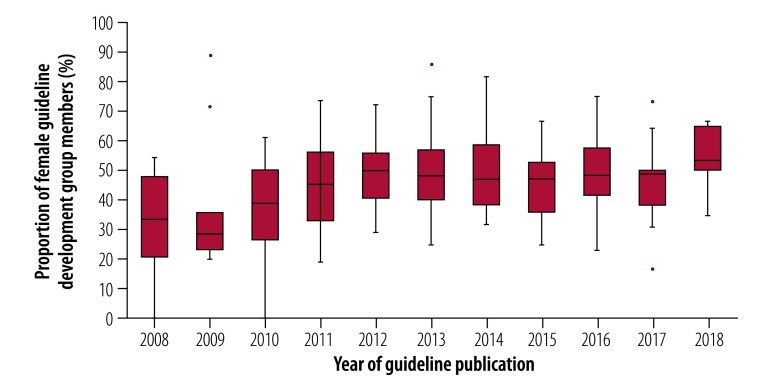
Proportion of WHO guideline development group members who were female, by year of guideline publication, 2008–2018

Notes: For each guideline, we determined and assigned the proportion of guideline development group members who were female as a value for that guideline. Hence, values below 50% represent groups where the proportion of members who were female was less than 50%. In the plot, the lines represent the medians, the boxes interquartile ranges, the error bars maximum and minimum, and the dots outliers.

For each guideline where a guideline development group was reported, we calculated the proportion of the members who were female. Fig. 1 depicts descriptive statistics for this indicator by year of guideline publication. The median proportion of group members who were female was 47.1% (IQR: 35.7–56.3). Within the guideline groups, the proportion of members who were female ranged from 0% to 88.9% (Table 2). 

**Table 2:** Gender distribution of WHO guideline development group members, 2008–2018

Year	No. of guidelines analysed	% of female members in guideline development group				No. (%) of guideline development groups									
		Median	Min.	Max.		≤ 10.0% female	10.1–20.0% female	20.1–30.0% female	30.1–40.0% female	40.1–50.0% female	50.1–60.0% female	60.1–70.0% female	70.1–80.0% female	80.1–90.0% female	90.1–100.0% female
2008	10	33.4	0.0	54.5		2 (20.0)	0 (0.0)	3 (30.0)	0 (0.0)	2 (20.0)	3 (30.0)	0 (0.0)	0 (0.0)	0 (0.0)	0 (0.0)
2009	11	28.6	20.0	88.9		0 (0.0)	2 (18.2)	4 (36.4)	3 (27.3)	0 (0.0)	0 (0.0)	0 (0.0)	1 (9.1)	1 (9.1)	0 (0.0)
2010	16	39.1	0.0	61.1		1 (6.3)	0 (0.0)	5 (31.3)	3 (18.8)	3 (18.8)	3 (18.8)	1 (6.3)	0 (0.0)	0 (0.0)	0 (0.0)
2011	28	45.4	19.0	73.7		0 (0.0)	1 (3.6)	3 (10.7)	8 (28.6)	6 (21.4)	8 (28.6)	1 (3.6)	1 (3.6)	0 (0.0)	0 (0.0)
2012	19	50.0	29.0	72.4		0 (0.0)	0 (0.0)	1 (5.3)	4 (21.1)	8 (42.1)	2 (10.5)	3 (15.8)	1 (5.3)	0 (0.0)	0 (0.0)
2013	21	48.3	25.0	85.7		0 (0.0)	0 (0.0)	3 (14.3)	3 (14.3)	7 (33.3)	6 (28.6)	0 (0.0)	1 (4.8)	1 (4.8)	0 (0.0)
2014	21	47.1	31.8	81.8		0 (0.0)	0 (0.0)	0 (0.0)	7 (33.3)	4 (19.0)	5 (23.8)	1 (4.8)	3 (14.3)	1 (4.8)	0 (0.0)
2015	18	47.1	25.0	66.7		0 (0.0)	0 (0.0)	2 (11.1)	5 (27.8)	4 (22.2)	4 (22.2)	3 (16.7)	0 (0.0)	0 (0.0)	0 (0.0)
2016	31	48.3	0.0	75.0		1 (3.2)	0 (0.0)	2 (6.5)	4 (12.9)	12 (38.7)	6 (19.5)	5 (16.1)	1 (3.2)	0 (0.0)	0 (0.0)
2017	22	48.8	16.7	73.7		0 (0.0)	1 (4.5)	0 (0.0)	5 (22.7)	12 (54.5)	1 (4.5)	1 (4.5)	2 (9.1)	0 (0.0)	0 (0.0)
2018	22	53.4	34.8	66.7		0 (0.0)	0 (0.0)	0 (0.0)	2 (9.1)	5 (22.7)	9 (40.9)	6 (27.3)	0 (0.0)	0 (0.0)	0 (0.0)
**Total**	**219**	**47.1**	**0.0**	**88.9**		**4 (1.8)**	**4 (1.8)**	**23 (10.5)**	**44 (20.1)**	**63 (28.8)**	**47 (21.5)**	**21 (9.6)**	**10 (4.6)**	**3 (1.4)**	**0 (0.0)**

WHO: World Health Organization.

Notes: For each guideline, we calculated the proportion of guideline development group members who were female (values range from 0.0% to 88.9%). For each year (2008–2018), we calculated the median proportion of the guideline development group who were female, for guidelines published in that year. We categorized the median proportions for each year into deciles. The table shows the number of guidelines per year, the median, minimum and maximum proportion of guideline development groups that are female and the decile distribution of the values.

Notably, for most years the median proportion of guideline development group members who were female was below 50.0%. Only 110 guidelines (50.2%) had a group composed of 40.1% to 60.0% females, 75 guidelines (34.2% of all guidelines) had ≤ 40% females in the guideline development group and 34 guidelines (15.5% of all guidelines) had > 60% female.

Across the years 2008–2018, there was wide variation in the gender balance of guideline development groups (Table 2). The median proportion of guideline development group members who were female was below 50% across most years. In 2008, 50.0% (5/10) of guidelines had 40.1‒60.0% females but in 2009 no guidelines fell in this range. Since 2016, 60.0% (45/75) of guidelines were 40.1‒60% female. In 2016 and 2017, approximately one-quarter of guideline development groups were composed of < 40% females (13/53), although this improved to 9.1% (2/22) in 2018.

A total of 131 guidelines specified at least one guideline development group chair or co-chair, constituting a total of 243 individuals. The gender imbalance of chairs was more marked, with 96 females (39.5%), 145 males (59.7% of all chairs) and 2 individuals (0.8%) of unknown gender.

### Other roles

A WHO steering group was specified in 225 guidelines, totalling 3721 individuals who were staff at WHO headquarters, regional offices and country offices. Among all WHO steering group members, 1637 (43.9%) were female and 2044 (54.9%) male. The median proportion of WHO steering group members who were female was 43.7% (IQR: 28.0–57.1). In total, 81 guidelines (36.0%) had a WHO steering group with a gender balance, 101 guidelines (44.9%) had a WHO steering group composed of ≤ 40.0% females, and 48 guidelines (21.3%) had a WHO steering group with > 60.0% females. 

A methodologist was specified in 98 guidelines, totalling 282 individuals: 179 (63.5%) were female, 102 (36.2%) male and 1 (0.4%) of unknown gender.

An external review group was specified in 155 guidelines, totalling 2355 individuals. Among all members, 1045 (44.4%) were female and 1279 (54.3%) male. The median proportion of members who were female was 44.4% (IQR: 33.3–56.7%). In total, 54 guidelines (34.8%) had an external review group composed of  40.1‒60.0% females, 68 guidelines (43.9) had < 40% females, and 33 guidelines (21.3%) had > 60.0% females.

## Discussion

We assessed the gender composition of guideline panels for all WHO guidelines published over an 11-year period. Overall, women were under-represented across most roles. While gender balance appeared to be improving in recent years, only half of the guidelines had a guideline development group composed of 40.1–60.0% female members, and more than one-third of the guidelines had a guideline development group composed of less than 40.0% female members. These findings suggest systematic, historical gender inequalities exist within an important domain of WHO’s global health leadership responsibilities.

Individuals involved in guideline development have different decision-making power and influence depending on their role. The WHO steering group is formed by the technical unit (department) responsible for the guideline, and is responsible for drafting the scope of the guideline and key questions, and selection and appointment of the guideline development group, methodologists and external review group, among other tasks.^16^ The guideline development group is the main decision-making entity, responsible for formulating recommendations through a consensus process, and have voting rights, if voting is required.^16^ The group chair is typically appointed by the WHO steering group, and accepted by the group, and facilitates the consensus process.^16^ The external review group peer reviews the final guideline; but are not responsible for modifying recommendations.^16^

Transformative leadership starts with a clear understanding of the current systems that perpetuate inequalities, unequal power dynamics and harmful social norms, roles and relationships.^3^ Calls for gender equality and representation in global health have grown in recent years as a result of global advocacy and shifting power structures.^17^^–^^19^ Capturing trends resulting from this momentum towards diversity and equality will serve the community well in maintaining positive change and providing necessary empirical evidence for further progress. This analysis can also serve to highlight the value of policy and practice protocols that privilege balanced representation. For example, the trends we found across WHO guidelines suggest that the requirement for gender parity stipulated in the *WHO handbook for guideline development* may have achieved a slight impact in improving the representation of women.

Overall, our findings demonstrate that gender composition of guideline panels has been slowly shifting towards improved parity in recent years. We note that gender is just one of many social stratifications that impact diversity. Achieving parity or balance in this domain may not be necessary nor sufficient to ensure that the perspectives of marginalized populations are included in decision-making. However, understanding the gender balance is an important first step to improving inclusivity in guideline development. While a growing evidence base exists on the current state of and action towards gender equality, less robust evidence is available on the complex geopolitics of global health leadership and the shifting power structures that define it. The debate around dismantling structures of power in global health that contribute to disparities has started and strengthening the evidence base to inform this debate will be pertinent. An important next step is to understand what an appropriate balance of representation and power may be across different geographical contexts.

Parity is a strategy to remedy imbalances resulting from structural inequalities, and achieving parity requires deliberate and directive organizational changes. For example, the United Nations System-wide Action Plan on Gender Equality and the Empowerment of Women provides clear guidance on strengthening institutional accountability related to gender mainstreaming and equal representation.^20^ Continued effort is needed to ensure that WHO’s commitment to gender balance in guideline development is consistently implemented. When people from diverse backgrounds – including different genders, cultures, ethnicities, and religions – join forces, they bring with them their own experiential knowledge that enriches discussions and promotes equality. Concerted efforts to improve diversity in teams or groups have had notable success across sectors. Research in management sciences has shown that scholarly contributions written by predominantly female author groups ask different questions and research different topics than their male counterparts.^21^ Women may enhance social perceptiveness (awareness of others’ reactions and understanding why they react in that manner) and collective intelligence during group activities, ultimately leading to more productive teams.^21^ Addressing systemic gender biases ultimately has the potential for transformational impact on “medical research, the accumulation of scientific knowledge, and the application of knowledge to practice,” as gender composition may influence the formation of research questions, medical practice and policy development.^22^

With the support of the WHO Guideline Review Committee, we had access to a comprehensive database of all WHO guidelines. A potential limitation of our analysis is the possibility that the gender assigned by the authors may not correspond to an individual’s gender identity. To mitigate this, we categorized individuals according to gender pronouns or titles used in guideline documents, or on their institutional websites or professional accounts. For people who identified as transgender, we used their preferred pronouns based on their current gender identity (< 1.0% of guideline contributors). We consider the possibility of misclassification to be low, and unlikely to bias the findings. 

We were interested to explore whether the scope or topic of the guideline was related to the gender composition of the guideline development group. However, given the diversity of topics and the overlapping nature of many guideline topics, we were not able to apply a mutually exclusive classification system. Future research exploring how the topic or target population of the guideline is related to the gender composition of groups would nonetheless be instructive.

The findings of gender imbalance among guideline development group chairs should be interpreted with some caution, as nearly half of all guidelines did not specify a chair. However, given the gender imbalances in guideline development group panels overall (from whom the chair is drawn), it appears unlikely that this finding is misleading. 

WHO’s 13th General Programme of Work outlines the strategic direction for 2019 to 2023, and specifically commits WHO “to the implementation of gender equality, equity and rights-based approaches to health that enhance participation, build resilience, and empower communities”^14^. The programme acknowledges that working effectively on gender equality and health equity will require WHO to reflect on its own policies and practice around inclusion, diversity and gender parity.^14^ Gender awareness is crucial to mitigate gender bias. Our analysis provides a starting point to reflect on and improve gender balance within one of WHO’s key normative functions. As demonstrated in our analysis, inequalities persist even where standards and policies call for gender balance. Attention can be shifted to improving the situation by strengthening accountability mechanisms and understanding the root causes. By addressing gender imbalances, everyone will benefit through more inclusive, more representative, and higher quality guidelines to improve health.

A first step is improved reporting of the composition of WHO guideline contributors throughout the guideline development process, from the proposal through to final publication. WHO steering groups should reflect on the balance of gender composition of all proposed contributors, and promote more even representation. Because inequalities may result from implicit biases,^23^^,^^24^ gender-sensitive training may be a useful measure for WHO staff involved in guideline development, to ensure that all qualified individuals (regardless of gender) can contribute to global health guidance. Further, a more transparent process of guideline development group selection may bring in fresh perspectives and increased opportunity for individuals from institutions with lower visibility or access to the global health landscape to contribute. While cultural shifts and organizational changes may be needed to support change, WHO could embrace a data-driven approach, setting targets and benchmarks to measure progress towards achieving better gender representation in the guideline development process.
