# Corrigendum

**DOI:** 10.2471/BLT.19.100819

**Published:** 2019-08-01

In: Bohren MA, Javadi D, Vogel JP. Gender balance in WHO panels for guidelines published from 2008 to 2018 Bull World Health Organ. 2019 Jul 1;97(7):477–85.

on page 479, Table 1 should read as follows:

**Table 1:** Individuals contributing to WHO guideline development, by gender and role in 219 guidelines published, 2008–2018

Role	No. (%) of individuals^a^			
	Total	Female	Male	Unknown gender
**Guideline development group member (including chair)**	4 912 (36.9)	2 241 (45.6)	2 606 (53.1)	65 (1.3)
**Guideline development group chair**	243 (1.8)	96 (39.5)	145 (59.7)	2 (0.8)
**Guideline methodologist**	282 (2.1)	179 (63.5)	102 (36.2)	1 (0.4)
**WHO steering group member**	3 721 (27.9)	1 637 (43.9)	2 044 (54.9)	40 (1.1)
WHO headquarters staff	3 035 (22.8)	1366 (45.0)	1640 (54.0)	29 (1.0)
WHO regional or country office staff	686 (5.1)	271 (39.5)	404 (58.9)	11 (1.6)
**External review group staff**	2 355 (17.7)	1 045 (44.4)	1 279 (54.3)	31 (1.3)
**Other^b^**	2 059 (15.4)	896 (43.5)	909 (44.1)	254 (12.3)
**Total**	**13 329 (100.0)**	**5 998 (45.0)**	**6 940 (52.1)**	**391 (2.9)**

WHO: World Health Organization.

^a^ Column percentages do not total 100.0% because of sub-categories (guideline development group chairs are also guideline development group members, WHO headquarters and WHO regional or country offices are part of WHO staff) overlapping with categories.

^b^ Please see Box 1 for roles included in this group.

Note: Of 230 guidelines identified, we excluded 11 guidelines from this analysis because they did not explicitly name panel members in the guideline development group.

